# Modeling of Dynamic Behavior of Carbon Fiber-Reinforced Polymer (CFRP) Composite under X-ray Radiation

**DOI:** 10.3390/ma11010143

**Published:** 2018-01-16

**Authors:** Kun Zhang, Wenhui Tang, Kunkun Fu

**Affiliations:** 1 College of Liberal Arts and Sciences, National University of Defense Technology, Changsha 410073, China; nudtzhangkun@163.com; 2 Centre for Advanced Materials Technology, School of Aerospace, Mechanical and Mechatronic Engineering, the University of Sydney, Camperdown, NSW 2006, Australia

**Keywords:** X-ray radiation, CFRP composite, dynamic behavior, BOI momentum, FEM code

## Abstract

Carbon fiber-reinforced polymer (CFRP) composites have been increasingly used in spacecraft applications. Spacecraft may encounter highenergy-density X-ray radiation in outer space that can cause severe damage. To protect spacecraft from such unexpected damage, it is essential to predict the dynamic behavior of CFRP composites under X-ray radiation. In this study, we developed an in-house three-dimensional explicit finite element (FEM) code to investigate the dynamic responses of CFRP composite under X-ray radiation for the first time, by incorporating a modified PUFF equation-of-state. First, the blow-off impulse (BOI) momentum of an aluminum panel was predicted by our FEM code and compared with an existing radiation experiment. Then, the FEM code was utilized to determine the dynamic behavior of a CFRP composite under various radiation conditions. It was found that the numerical result was comparable with the experimental one. Furthermore, the CFRP composite was more effective than the aluminum panel in reducing radiation-induced pressure and BOI momentum. The numerical results also revealed that a 1 keV X-ray led to vaporization of surface materials and a high-magnitude compressive stress wave, whereas a low-magnitude stress wave was generated with no surface vaporization when a 3 keV X-ray was applied.

## 1. Introduction

Carbon fiber-reinforced plastic (CFRP) composites exhibit excellent mechanical properties such as high strength, high stiffness, high impact resistance, light weight, and low thermal expansion, conferring considerable potential on such composites for spacecraft applications [1,2,3,4,5]. Generally, the external wall of a spacecraft is covered by a CFRP shell to provide shielding protection for the interior of the vehicle. In addition to providing superior thermal ablation protection during the re-entry into the atmosphere [6,7], the design of a CFRP protection shell also needs to consider the potential damage induced by radiation [8,9,10,11,12,13,14]. In outer space, spacecraft may encounter highenergy-density X-ray radiation induced by a nuclear explosion [15,16,17]. When the X-ray irradiates on the CFRP composites, it is instantaneously transformed into internal energy. If the specific deposition energy is sufficiently high, the surface of the CFRP composite may vaporize. The vaporized material expands violently and generates a blow-off impulse (BOI) momentum, resulting in a compressive stress wave propagating in the residual materials. At the same time, the high internal energy induces rapid thermal expansion in the material, leading to a thermally induced stress wave. These two stress waves integrate, propagating in the residual CFRP composite, posing a threat to the structural safety of the spacecraft. For instance, a previous study [17] reported that X-ray radiation by a nuclear explosion significantly downgraded the performance of a satellite shield. Therefore, in order to enhance the survivability of spacecraft, it is essential to quantitatively predict the dynamic behavior of CFRP composites under X-ray radiation.

Over decades, the mechanism of X-rays interacting with target materials has been extensively investigated [18,19,20,21,22,23]. X-ray photons interact with atoms of a target material, inducing an upheaval of specific energy in the target materials. Once the specific energy distribution is known, BOI-induced stress and thermalexpansion-induced stress can be calculated by several one-dimensional theoretical models [23]. For the computation model of the BOI-induced stress, the BOI momentum and compressive stress can be evaluated by either the Whitener model, the BBAY model, or the modified BBAY model. In ref. [24], these three models were compared by assuming uniform and exponential internal specific energy distribution. In comparisons of the nuclear experiment using these three models, however, a large discrepancy between the theoretical prediction and the experimental result was found. Because nuclear explosion experimentation is extremely expensive, experimental investigation of radiation on materials is rare. The Z-pinch device has been used experimentally to produce a soft X-ray radiation impulse in metal, meteorite and planetary materials [20,21,22]. The pressure, BOI momentum, and momentum-coupling coefficient were measured to study the dynamic behavior of those substances. Regarding thermalexpansion-induced stress, Langley et al. [23] discussed the mechanism of thermal expansion in materials caused by an X-ray. Morland et al. [25] provided an analytical solution for the thermalexpansion-induced stress in a one-layer isotropic material. Furthermore, Gascoigne et al. [26] improved the analytical solution to assess the thermalexpansion-induced stress in multilayered target material. To simplify the theoretical analysis in the above research, the targets were assumed to be elastic and the solutions were only applied in onedimension. Recently, numerical modeling has become increasingly attractive compared to expensive experiments and simplified analytical solutions, due to its advantages such as low cost, time saving, and ease of modeling complex systems. Several numerical models [18,22,27] have been developed to evaluate the dynamic performance of metallic materials. For example, Cost et al. [27] utilized the Whitener model to determine BOI momentum, and subsequently, the BOI momentum was used as an external loading to predict the dynamic response of an aluminum target by a traditional hydrocode program. In that work [27], however, thermalexpansion-induced stress was neglected. Later, Remo et al. [22] and Asay et al. [18] utilized a CTH program and an ALEGRA program developed by Sandia National Laboratories to investigate the dynamic behavior of a metal target. In contrast to the work in ref. [27], the SESAME equation-of-state (EOS) package was used to calculate the sublimation, the phase change of vaporized materials, and the stress-wave propagation. 

To the best of our knowledge, existing studies have focused mainly on X-ray radiation on metal, meteorite, and planetary materials. The investigation of CFRP composite radiation is rare. More recently, Huang et al. [28] for the first time developed a two-dimensional finite element (FE) model incorporating PUFF EOS to simulate the dynamic behavior of a CFRP target irradiated by an X-ray. Unlike metal and meteorite material, CFRP material is anisotropic. The following points need to be considered to simulate CFRP material accurately: (a) anisotropy of stiffness, Poisson ratio, yield strength, and other properties; (b) coupling between hydrostatic pressure and deviatoric stress; and (c) the non-linear relationship between pressure and volumetric strain in the high-pressure phase. Considering these three points, Anderson et al. [29,30] successfully developed a numerical model to simulate stress-wave propagation in an anisotropic material. Clegg et al. [31] and Riedel et al. [32] used a FE model to examine the ballistic protection performance of composite materials. Extensive follow-up experiments and numerical models [33,34,35,36] investigated the dynamic behavior of CFRP materials. In those studies, the CFRP materials were in a compression condition, and hence Grüneisen or simplified polynomial forms of EOS were suitable for describing the non-linear relationship between the pressure and volumetric strain. However, for X-ray radiation on CFRP composites, the EOS should be modified to incorporate the effects of sublimation and expansion in orthotropic material. 

This study aimed to develop a three-dimensional FEM program to examine the dynamic response of a CFRP target irradiated by X-ray. A modified PUFF EOS was introduced to describe the phase change and sublimation of an anisotropic CFRP material. The rest of the paper is organized as follows: in Section 2, theoretical descriptions of the radiation mechanism and the constitutive model of the CFRP material are introduced. Section 3 describes the implementation of the in-house FEM program. In Section 4, the presented FEM program is validated by an existing radiation experiment on an aluminum target. After the validation, the dynamic behavior of a CFRP target irradiated by X-rays with different blackbody temperatures is examined. The main conclusions are drawn in Section 5.

## 2. Mechanism of Stress-Wave Induced by X-ray Pulse

### 2.1. X-ray Radiation and Energy Deposition

**Figure 1:**
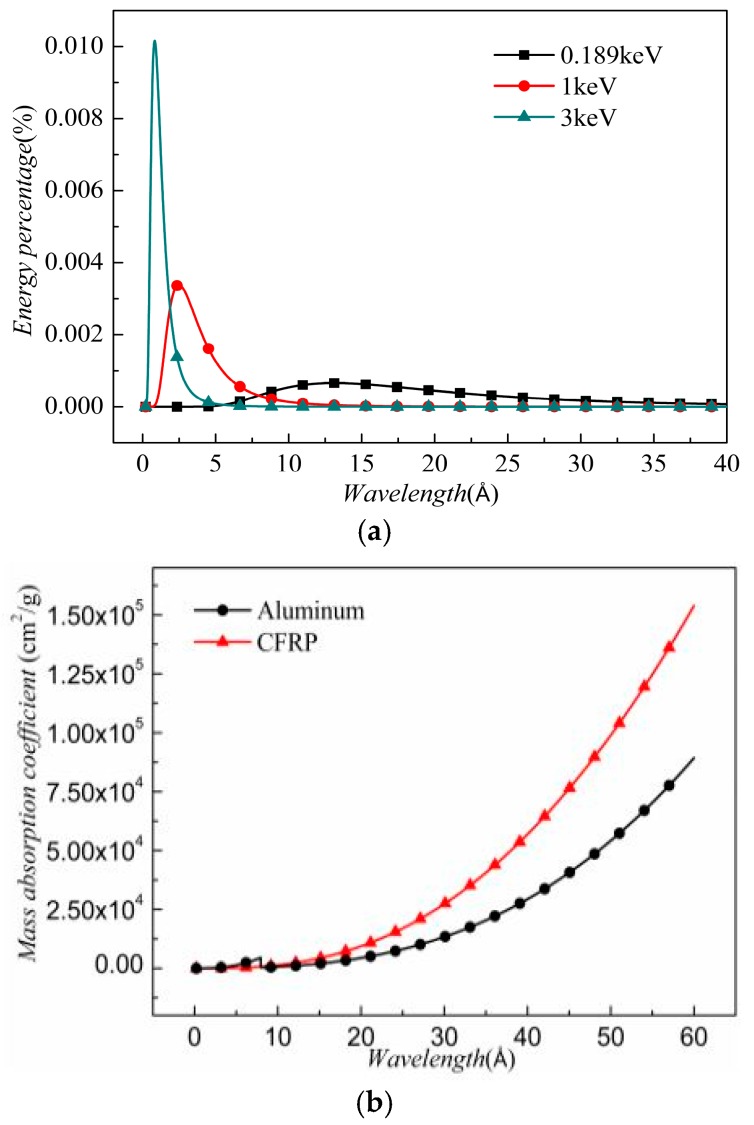
(**a**) Energy percentage; and (**b**) mass absorption coefficient.

X-ray generated by a nuclear explosion is a typical electromagnetic wave with a continuous wavelength distribution. Based on the blackbody radiation model [37], the monochromatic emissivity of an X-ray source with temperature *T* and wavelength *λ* is expressed as:

(1) $$f(\lambda ,T)=\frac{c_{1}}{\lambda }\frac{1}{\exp(\frac{c_{2}}{\lambda T})-1}=\frac{2\pi hc^{2}}{\lambda^{5}}\frac{1}{\exp(\frac{hc}{\lambda kT})-1}$$

where *c*_1_ and *c*_2_ are the radiation constants; $c_{1}=3.7435\times 10^{-12}\;J\cdot {\mathrm{cm}}^{2}/s$; $c_{2}=1.439\;\mathrm{cm}\cdot K$; *h* is the Planck constant; $h=6.62\times 10^{-34}\;J\cdot s$; *c* is the light speed; *k* is the Boltzmann constant; and $k=1.38\times 10^{-23}\;J/K$. The continuous wavelength from 2–60 Å is divided into 5000 equal parts to ensure the dispersion accuracy. The interval is defined as Δλ. For a photon with the wavelength $\lambda_{i}$ (*i* = 1–5000), the percentage of incident energy flux in each *λ* is given by:

(2) $$w_{i}(\lambda )=\frac{f(\lambda_{i},T)\Delta \lambda_{i}}{\int_{a}^{b}f(\lambda_{i},T)d\lambda }=\frac{f(\lambda_{i},T)\Delta \lambda_{i}}{\sigma T^{4}}$$

where *σ* is the Stefan–Boltzmann constant; and $\sigma =5.67\times 10^{-12}\;J/\left({\mathrm{cm}}^{2}\cdot s\cdot K^{4}\right)$. The physical mechanism of the X-ray interacting with the target is the photoelectric effect and the Compton scattering effect [27]. When an X-ray pulse with an initial fluence $\Phi_{0}$ radiates onto the target, the absorbed energy is characterized as an exponential form in which the energy fluence Φ(x) declines with the depth *x*. For a one-dimensional problem, Φ(x) is given by:

(3) $$\Phi (x)=\sum_{1}^{i}w_{i}\Phi_{0}\exp\left[-\rho_{0}\mu_{i}(\lambda )x\right]\approx \Phi_{0}\exp(-\rho_{0}\mu_{eff}x)$$

where $\rho_{0}$ is the initial density; and $\mu_{i}(\lambda )$ is the mass absorption coefficient related to the mass percentage of the material and the photon wavelength. Here, aluminum and CFRP are two target materials. The $\mu_{i}(\lambda )$ of the aluminum and CFRP targets are calculated from the database in [38] as shown in Figure 1a. 

The mass absorption coefficient increases with the increase in wavelength, and the mass absorption coefficient of the CFRP is greater than that of the aluminum. It is worthwhile noting that a bump is seen at 7.95 Å, indicating the absorption edge of aluminum. $\mu_{eff}$ is the effective absorption coefficient, which approximates the relation in Equation (3). $\mu_{eff}$ is defined as:

(4) $$\mu_{eff}=\sum_{1}^{i}w_{i}\mu_{i}$$

$\mu_{eff}$ reflects the energy absorption capacity of the target materials. In this work, *kT* = 0.189 and *kT* = 1 keV are two representatives for the soft X-ray, whereas *kT* = 3 keV is a typical hard X-ray. The distribution of the energy percentage of these three X-rays is shown in Figure 1b. In a hard X-ray, the photons with a shorter wavelength possess most of the energy. In a soft X-ray, the peak value of the energy percentage is lower than that in a hard X-ray. Using Equation (4), the $\mu_{eff}$ are obtained. Under a 0.189 keV X-ray, the $\mu_{eff}$ in aluminum and CFRP are 7885 and 15,363 cm^2^/g, respectively. For a CFRP target, the $\mu_{eff}$ under 1 keV X-ray and 3 keV X-ray are found to be 319 and 15 cm^2^/g, respectively. These parameters are used in the following numerical simulation.

The attenuated photon energy deposits into the material and converts into internal energy. From a depth from $x_{1}$ to $x_{1}+\Delta x$ on a unit area, the specific internal energy e(x) is given by:

(5) $$e(x_{1})=\frac{\Phi (x_{1})-\Phi (x_{1}+\Delta x)}{\rho_{0}\Delta x}=\frac{\Phi_{0}\exp(-\rho_{0}\mu_{eff}x_{1})(1-\exp(-\rho_{0}\mu_{eff}\Delta x))}{\rho_{0}\Delta x}\approx \mu_{eff}\Phi_{0}\exp(-\rho_{0}\mu_{eff}x_{1})$$

Equation (5) is applicable in one-dimensional model. For the three-dimensional model, an algorithm was developed in our previous work [39] to calculate the energy deposition.

### 2.2. Stress Induced by Blow-Off Impulse (BOI) and Thermal Expansion

As already stated, X-ray radiation leads to exponentially distributed specific internal energy in materials. The rate of attenuation of X-ray energy in the materials is directly related to $\mu_{eff}$. When the $\mu_{eff}$ is high, the rate of attenuation of X-ray energy is high. Thus, most X-ray energy deposits onto the thin surface layer of the materials, subsequently transforming into internal energy. When the specific internal energy exceeds the threshold of vaporization energy $e_{0}$, the surface materials on the radiated area vaporize. Generally, a phase change is completed almost instantaneously due to the short duration and high energy of an X-ray pulse. Due to this fact, in our model no liquidation or further ionization is considered, for simplicity. The vaporized materials are in a state with high specific internal energy and high pressure, whereas their density is still close to that of the initial solid state. Then, the vaporized materials expand rapidly with a decrease in the pressure and density. Based on the conservation of momentum, the vaporized materials impose a recoil momentum on the residual materials, leading to the propagation of a compressive stress in the materials. The stress is usually characterized as a form of triangular wave with a sharp rise and slow attenuation. The BOI momentum can be calculated by the modified BBAY model as follows:

(6) $$I=\alpha \sqrt{2}{\left\{\int_{0}^{x_{0}}\left[e(x)-e_{0}(1+\ln(\frac{e(x)}{e_{0}})\right]\rho^{2}xdx\right\}}^{2}$$

where $1\leq \alpha \leq \sqrt{2}$. $x_{0}$ denotes the thickness of the vaporization layer. The following equation should be satisfied:

(7) $$e(x_{0})\approx \mu_{eff}\Phi_{0}\exp(-\rho_{0}\mu_{eff}x_{0})=e_{0}$$

If $\mu_{eff}\Phi_{0}\gg e_{0}$, the solution of $x_{0}$ in Equation (7) exists and thus, vaporization occurs.

The compressive stress induced by the BOI is characterized as an average stress $\sigma_{s}$ with a temporal pulse width $\tau_{s}$:

(8) $$\sigma =\frac{I}{\tau_{s}}$$

**Figure 2:**
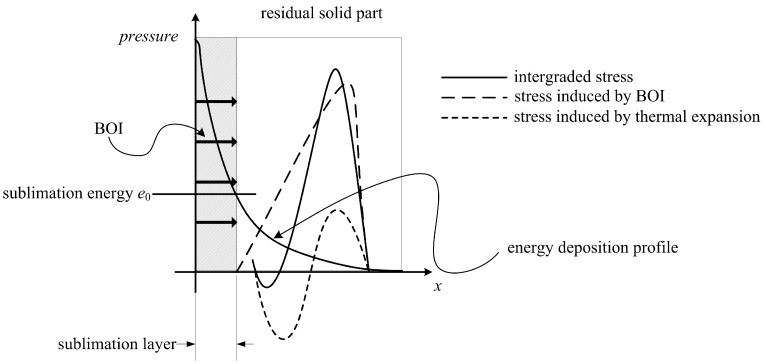
Schematic of the integrated stress wave.

If Equation (7) has no solution, this indicates that the specific internal energy in the residual materials does not reach the vaporization threshold. Therefore, the energy in the residual materials is still exponentially distributed. The non-uniform energy deposition generates a non-uniform, thermalexpansion-induced stress. Governed by the equation of motion, the stress propagates in the materials. Except for the stress wave propagating to the back surface, this compressive stress also propagates to the free front surface, reflected by the front surface as a reversely symmetrical tensile stress. Because no mass removal is involved, the momentum of the target materials should remain zero. Therefore, the thermalexpansion-induced stress wave propagating to the back surface is followed by a symmetrical rarefaction wave. The BOI-induced stress and thermalexpansion-induced stress integrate, as illustrated in Figure 2. 

The integrated stress wave propagates, attenuates and reflects in the materials. The physical process of X-ray radiation on CFRP composites is complex, and, therefore, it is nearly impossible to derive an analytical solution to determine the dynamic behavior of the materials, especially in three-dimensional space. Moreover, it is worth noting that if the reflected tensile stress meets the maximum tensile strength criterion, as defined in Equation (9), fracture occurs in the materials and produces front-surface spallation.

(9) $$\sigma_{ij}\geq \sigma_{ij}^{fracture}$$

### 2.3. Elastic Constitutive Model

In this section, a constitutive relation of CFRP materials is introduced. The CFRP material is regarded as a homogenized orthotropic material. For elastic deformation, the generalized Hook’s law in principal axes is represented in Equation (10):

(10) $$\left(\begin{array}{c} \sigma_{11}\\ \sigma_{22}\\ \sigma_{33}\\ \sigma_{12}\\ \sigma_{13}\\ \sigma_{23} \end{array}\right)=\left(\begin{array}{cccccc} c_{11} & c_{12} & c_{13} & 0 & 0 & 0\\ & c_{22} & c_{23} & 0 & 0 & 0\\ & & c_{33} & 0 & 0 & 0\\ & symm & & c_{44} & 0 & 0\\ & & & & c_{55} & 0\\ & & & & & c_{66} \end{array}\right)\left(\begin{array}{c} \varepsilon_{11}\\ \varepsilon_{22}\\ \varepsilon_{33}\\ \varepsilon_{12}\\ \varepsilon_{13}\\ \varepsilon_{23} \end{array}\right)$$

where $\sigma_{ij}$, $\varepsilon_{ij}$, and $c_{ij}$ are the stress, strain, and stiffness tensors in principal axes, respectively (*i* = 1–3 and *j* = 1–3). Due to the symmetry, the stiffness tensor matrix only has nine independent parameters. The strain tensor can be partitioned into volumetric strain $\theta =\varepsilon_{11}+\varepsilon_{22}+\varepsilon_{33}$ and deviatoric strain $\sigma_{ij}^{d}$ as:

(11) $$\varepsilon_{ij}=\frac{\theta }{3}\delta_{ij}+\varepsilon_{ij}^{d}$$

where $\delta_{ij}$ denotes the Kronecker delta function. The stress tensor is decomposed into hydrostatic pressure *p* and deviatoric stress $s_{ij}$, which is expressed as:

(12) $$\sigma_{ij}=-p\delta_{ij}+s_{ij}$$

Substituting Equations (10) and (11) into Equation (12), the relation between *p* and $\varepsilon_{ij}$ is obtained:

(13) $$\begin{array}{ll} p= & -(c_{11}+c_{22}+c_{33}+2c_{12}+2c_{13}+2c_{23})\frac{\theta }{9}\\ & -(c_{11}+c_{12}+c_{13})\frac{\varepsilon_{11}^{d}}{3}-(c_{21}+c_{22}+c_{23})\frac{\varepsilon_{22}^{d}}{3}-(c_{31}+c_{32}+c_{33})\frac{\varepsilon_{33}^{d}}{3} \end{array}$$

The effective bulk modulus $K^{\prime }$ is defined as:

(14) $$K^{\prime }=\frac{\left(c_{11}+c_{22}+c_{33}+2c_{12}+2c_{13}+2c_{23}\right)}{9}$$

### 2.4. Plastic Constitutive Model

In CFRP material, the development of cracking and delamination are considered irreversible deformations. From a macroscale viewpoint, this irreversible deformation is similar to the irreversible plastic deformation in ductile metallic materials. Correspondingly, several plasticity models [40,41] have been used to simulate irreversible failure in CFRP composites. The yield criterion f is indicated as a totalstress-based nine-parameter quadratic form to assess the stress state (elastic or plastic):

(15) $$\begin{array}{ll} f(\sigma_{ij})= & a_{11}\sigma_{11}^{2}+a_{22}\sigma_{22}^{2}+a_{33}\sigma_{33}^{2}+2a_{12}\sigma_{11}\sigma_{22}+2a_{23}\sigma_{22}\sigma_{33}\\ & +2a_{13}\sigma_{11}\sigma_{33}+2a_{44}\sigma_{23}^{2}+2a_{55}\sigma_{31}^{2}+2a_{66}\sigma_{12}^{2}\leq k(\varepsilon^{p}) \end{array}$$

where $k(\varepsilon^{p})$ is the state variable correlated with effective plastic strain. The yield surface in normal stress space is a closed convex ellipsoid. If $f(\sigma_{ij})<k(\varepsilon^{p})$, the material is in an elastic state and the stress tensor is still in the yield surface. If $f(\sigma_{ij})\geq k(\varepsilon^{p})$, which means the stress tensor exceeds the yield surface and that is prohibited. $k(\varepsilon^{p})$ is adjusted to ensure that the stress tensor lies on the yield surface. Based on the normality and flow rule, the plastic strain increment scales linearly with the associated gradient of the yield criterion, defined as:

(16) $$d\varepsilon_{ij}^{p}=\beta \frac{\partial F}{\partial \sigma_{ij}}$$

where β is the plastic strain multiplier. According to plastic increment theory, the increments of stress and elastic strain satisfy the generalized Hooke’s law. Equation (10) is differentiated and rewritten in the tensor form:

(17) $$d[\sigma ]=[C]d[\varepsilon^{e}]=[C]d([\varepsilon ]-[\varepsilon^{p}])$$

where d[σ] presents the increment of stress, $d[\varepsilon^{e}]$ denotes the increment of elastic strain and d[ε] is the increment of strain which is calculated by the node motion. After obtaining the d[σ], we can update the stress tensor [σ] and $k(\varepsilon^{p})$. The increment of *p* can be expressed from the differential of Equations (11) and (16), which is shown as below:

(18) $$\begin{array}{ll} dp= & -(c_{11}+c_{22}+c_{33}+2c_{12}+2c_{13}+2c_{23})\frac{d\theta }{9}\\ & -(c_{11}+c_{12}+c_{13})\frac{d\varepsilon_{11}^{d}}{3}-(c_{21}+c_{22}+c_{23})\frac{d\varepsilon_{22}^{d}}{3}-(c_{31}+c_{32}+c_{33})\frac{d\varepsilon_{33}^{d}}{3}\\ & +(c_{11}+c_{12}+c_{13})\frac{d\varepsilon_{11}^{p}}{3}+(c_{21}+c_{22}+c_{23})\frac{d\varepsilon_{22}^{p}}{3}+(c_{31}+c_{32}+c_{33})\frac{d\varepsilon_{33}^{p}}{3} \end{array}$$

### 2.5. Modified Equation-of-State (EOS)

Experimental results [22] specific volume is non-linear. Therefore, an EOS is required to calculate this non-linear relationship and, furthermore, the phase change and thermal expansion caused by X-ray radiation should also be considered. For instance, to simulate the sublimation process in a CFRP material, Liu et al. [42] proposed using the Jones–Wilkins–Lee EOS to study the phase change and BOI caused by a lightning strike. In our study, a modified PUFF EOS was adopted and combined with the Grüneisen EOS to describe the physical process of X-ray radiation on CFRP materials. The Grüneisen in the compression zone of a solid target was expressed as:

(19) $$p=p_{H}(\upsilon )+\rho_{0}\Gamma_{0}(e-e_{H})=\frac{\rho_{0}c_{0}^{2}(1-\upsilon /\upsilon_{0})}{\left[1-s{\left(1-\upsilon /\upsilon_{0}\right)}^{2}\right]}+\rho_{0}\Gamma_{0}(e-e_{H})$$

For the thermal dilation of the solid and phase change zone, the following PUFF EOS was used:

(20) $$p=\rho_{0}[\gamma -1+(\Gamma_{0}-\gamma +1)\sqrt{\frac{\rho }{\rho_{0}}}][e-e_{0}[1-\exp[N\frac{\rho }{\rho_{0}}(1-\frac{\rho }{\rho_{0}})]]]$$

where $p_{H}$ and $e_{H}$ present the Hugoniot pressure and Hugoniot energy, respectively; $c_{0}$ and s are the Hugoniot parameters; ρ denotes the current density; υ=1/ρ denotes the specific volume; $\Gamma_{0}$ denotes the Grüneisen parameter, $N=c_{0}^{2}/\Gamma_{0}e_{0}$; and *γ* is the specific heat ratio of vaporized gas. The PUFF EOS has been widely used to describe a mixture of gas state and solid state, with several resulting advantages compared to other EOS, such as JWL: (a) joining smoothly with Grüneisen EOS at $\rho_{0}$; (b) sublimation energy can be considered; (c) PUFF EOS can be used well to describe both the mechanical performance of solid phase and the expansion of gas phase. Hence, we incorporated PUFF EOS to simulate the sublimation and thermal expansion of the CFRP composite under X-ray radiation.

The reduced density is defined as $\mu =\left(\rho -\rho_{0}\right)/\rho_{0}=\left(\upsilon -\upsilon_{0}\right)/\upsilon =-\theta$. The polynomial Taylor expansion of Equations (19) and (20) can be rewritten as:

(21) $$p=-A_{1}\theta +(A_{2}-\frac{\Gamma_{0}}{2}A_{1})\theta^{2}-(A_{3}-\frac{\Gamma_{0}}{2}A_{2})\theta^{3}+(1-\theta )\rho_{0}\Gamma_{0}e$$

(22) $$p=-B_{1}\theta +B_{2}\theta^{2}-B_{3}\theta^{3}+[\rho_{0}\Gamma_{0}-\frac{3\rho_{0}\Gamma_{0}}{2}\theta +\frac{\rho_{0}(\gamma -1)\theta }{2}]e$$

where

$$\left\{ \begin{array}{l} A_{1}=\rho c_{0}^{2}\\ A_{2}=A_{1}(2s-1)\\ A_{3}=A_{1}(3s^{2}-4s+1)\\ B_{1}=\rho c_{0}^{2}\\ B_{2}=-B_{1}/2-\left(\gamma -1\right)/2\Gamma_{0}B_{1}+B_{1}/2N\\ B_{3}=5B_{1}/24+5(\gamma -1)B_{1}/8\Gamma_{0}-(5/4+\left(\gamma -1\right)/4\Gamma_{0})B_{1}N+1/6B_{1}N^{2} \end{array}\right.$$

These constants are derived from experimental data fitting. Due to the implementation of incremental plastic theory, the EOS requires an incremental form to be calculated at each time step, which is denoted as:

(23) $$p=-A_{1}\theta +(A_{2}-\frac{\Gamma_{0}}{2}A_{1})\theta^{2}-(A_{3}-\frac{\Gamma_{0}}{2}A_{2})\theta^{3}+(1-\theta )\rho_{0}\Gamma_{0}e$$

(24) $$\begin{array}{ll} dp= & -B_{1}d\theta +2B_{2}\theta d\theta -3B_{3}\theta^{2}d\theta^{3}\\ & +[\rho_{0}\Gamma_{0}-\frac{3\rho_{0}\Gamma_{0}}{2}\theta +\frac{\rho_{0}(\gamma -1)\theta }{2}]de-[\frac{3\rho_{0}\Gamma_{0}}{2}-\frac{\rho_{0}(\gamma -1)}{2}]ed\theta \end{array}$$

Equations (19)–(24) are still traditional EOS, which are suitable for describing the rapid sublimation of an isotropic material. For orthotropic materials, however, the traditional EOS must be modified. The elastic terms in Equations (23) and (24) are replaced by Equation (12), and the modified equations are given by:

(25) $$\begin{array}{ll} dp= & -(c_{11}+c_{22}+c_{33}+2c_{12}+2c_{13}+2c_{23})\frac{d\theta }{9}\\ & -(c_{11}+c_{12}+c_{13})\frac{d\varepsilon_{11}^{d}}{3}-(c_{21}+c_{22}+c_{23})\frac{d\varepsilon_{22}^{d}}{3}-(c_{31}+c_{32}+c_{33})\frac{d\varepsilon_{33}^{d}}{3}\\ & +(c_{11}+c_{12}+c_{13})\frac{d\varepsilon_{11}^{p}}{3}+(c_{21}+c_{22}+c_{23})\frac{d\varepsilon_{22}^{p}}{3}+(c_{31}+c_{32}+c_{33})\frac{d\varepsilon_{33}^{p}}{3}\\ & +2(A_{2}-\frac{\Gamma_{0}}{2}A_{1})\theta d\theta -3(A_{3}-\frac{\Gamma_{0}}{2}A_{2})\theta^{2}d\theta +(1-\theta )\rho_{0}\Gamma_{0}de-\rho_{0}\Gamma_{0}ed\theta \end{array}$$

(26) $$\begin{array}{ll} dp= & -(c_{11}+c_{22}+c_{33}+2c_{12}+2c_{13}+2c_{23})\frac{d\theta }{9}\\ & -(c_{11}+c_{12}+c_{13})\frac{d\varepsilon_{11}^{d}}{3}-(c_{21}+c_{22}+c_{23})\frac{d\varepsilon_{22}^{d}}{3}-(c_{31}+c_{32}+c_{33})\frac{d\varepsilon_{33}^{d}}{3}\\ & +(c_{11}+c_{12}+c_{13})\frac{d\varepsilon_{11}^{p}}{3}+(c_{21}+c_{22}+c_{23})\frac{d\varepsilon_{22}^{p}}{3}+(c_{31}+c_{32}+c_{33})\frac{d\varepsilon_{33}^{p}}{3}\\ & +2B_{2}\theta d\theta -3B_{3}\theta^{2}d\theta^{3}+[\rho_{0}\Gamma_{0}-\frac{3\rho_{0}\Gamma_{0}}{2}\theta +\frac{\rho_{0}(\gamma -1)\theta }{2}]de-[\frac{3\rho_{0}\Gamma_{0}}{2}-\frac{\rho_{0}(\gamma -1)}{2}]ed\theta \end{array}$$

It can be seen that the modified EOS are related to both the high-order terms of volumetric strain and the energy terms. The pressure is related to the deviatoric strain and the plastic strain. Several studies [3,43,44] have successfully applied the aforementioned modified EOS model to analyze the dynamic behavior of CFRP material under hypervelocity impact. In this paper, the modified EOS model was incorporated in our FEM program to analyze the dynamic behavior of the CFRP composite under X-ray radiation. The thermal expansion of the CFRP composite was described by the modified PUFF equation, i.e., Equation (26), and the compressed solid part was denoted by the modified Grüneisen EOS in Equation (25). For the sublimation phase of the CFRP material, the PUFF EOS in Equation (26) degenerates to its original form in Equation (24) because no deviatoric and plastic strain exist in the vaporized gas.

## 3. Finite Element Model (FEM)

### 3.1. In-House FEM Code Implementation

**Figure 3:**
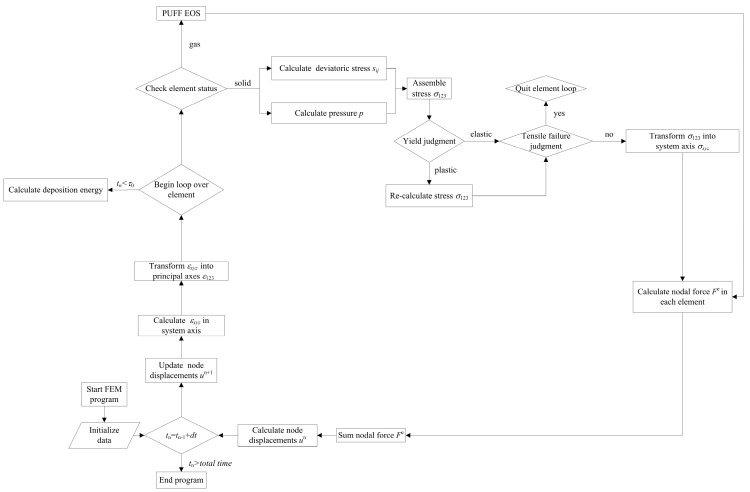
Flow chart of the finite element model (FEM) program.

Using the theoretical relation described in Section 2, an in-house Lagrangian explicit FEM program was developed here to investigate the dynamic responses of CFRP composites under X-ray radiation. A brief flowchart of the FEM program is shown in Figure 3.

First, the material parameters and initial conditions are input. The displacements of the nodes are updated by the equation of motions in the system axes to obtain the strain tensor. Then, the strain tensor is transformed into a principal coordinate. The state of the elements is checked to examine the gas element and solid elements. Correspondingly, the PUFF EOS is used to describe the gas elements and the elastic–plastic constitutive laws are used to determine the nodal force and displacement of solid elements at each time step. Before the end of the X-ray radiation, the energy deposition in each time step is calculated as the external loading. Finally, the stress tensor in all elements must be transformed back to the system coordinate to prepare for the next round of the loop. The coordinate transformation algorithm was detailed in [29]. The FEM calculation ends when the total time is reached.

### 3.2. Geometry and Boundary Conditions

**Figure 4:**
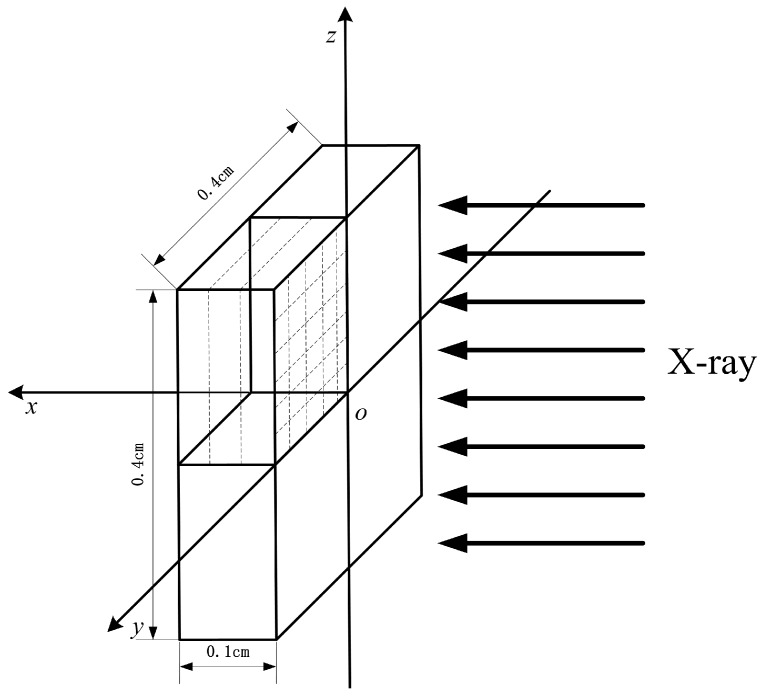
FE model of X-ray radiation on a carbon fiber-reinforced polymer (CFRP) composite.

A three-dimensional CFRP model was established with the geometry of 0.1 × 0.4 × 0.4 cm^3^. As shown in Figure 4, lamina stack direction, warp direction, and weft direction are parallel to the *x*, *y*, and *z* directions, respectively, to skip the initial coordinate transformation.

An X-ray is radiated on the CFRP model in the *x*-direction. The FE model was meshed with hexahedron elements of uniform size. Before the FE analysis, mesh sensitivity was conducted to establish a balance between accuracy and computational efficiency. The BOI momentum was determined using the FE model with different mesh size. We found that when reducing the mesh size, BOI momentum gradually increased to a constant value, whereas the run-time significantly increased. To balance the computational efficiency and accuracy, the convergent mesh size of 0.001 × 0.008 × 0.008 cm^3^ was used. Free boundary conditions were applied on all surfaces to simulate the stationary state of the CFRP target. To save computational time, one-quarter of the target was used in the FE model with a symmetrical boundary condition.

### 3.3. Material Properties

In our FE model, a unidirectional CFRP composite with the volume fraction of 52% was the focus. The lay-up sequence was [0/45/-45/-45/45/0]. The carbon fibers were Tenax UMS2526 and the matrices were epoxy resins (Krempel BD system) cured at 120 °C. The material properties were derived from experimental work [44] as listed in Table 1.

To validate the FEM program, the numerical results were compared with an existing experiment in which the target material was aluminum [22]. Aluminum is an isotropic elastic–plastic material, and its yield behavior follows the Von Mises yield criterion defined as:

(27) $$f(\sigma_{ij})=\sqrt{\frac{{\left(\sigma_{11}-\sigma_{22}\right)}^{2}+{\left(\sigma_{22}-\sigma_{33}\right)}^{2}+{\left(\sigma_{33}-\sigma_{11}\right)}^{2}+6(\sigma_{12}^{2}+\sigma_{13}^{2}+\sigma_{23}^{2})}{6}}\leq k.$$

The isotropic material can be regarded as a special case of an anisotropic material in which the Young’s modulus *E*, shear modulus *G*, and Poisson’s ratio *v* are the same in every direction. The effective bulk module $K^{\prime }$ in Equation (14) is equal to the traditional definition of bulk module $K=\rho_{0}c_{0}^{2}$:

(28) $$K^{\prime }=\frac{\left(c_{11}+c_{22}+c_{33}+2c_{12}+2c_{13}+2c_{23}\right)}{9}=\frac{E}{3(1-2v)}=\rho_{0}c_{0}^{2}.$$

The plastic strain term and deviatoric terms vanished because the traces of plastic strain and deviatoric strain tensors were zero [45]. The coordinate transformation matrix was the identity matrix. The constitutive relationship and EOS degenerated to the traditional condition automatically. The aluminum parameters were selected from the literature [40] and are listed in Table 2. It should be noticed that all parameters selected in Table 1 and Table 2 are obtained at a high strain rate. The strain-rate effect is neglected for simplicity.

## 4. Results and Discussion 

### 4.1. FEM Program Validation

**Figure 5:**
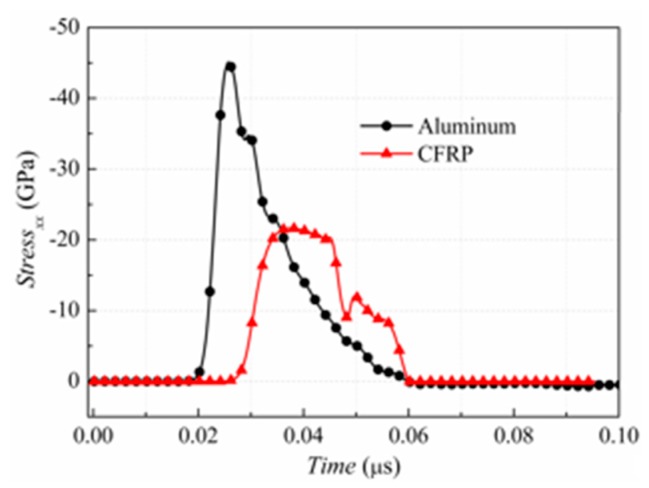
Stress history of CFRP and aluminium panels.

For the validate experiment, the target material was an aluminum panel with the thickness *H* of 1 mm. The X-ray blackbody temperature was 0.189 keV. The energy fluence $\Phi_{0}$ was 1710 J/cm^2^ and the duration of the pulse $\tau_{0}$ was 0.0066 μs. The X-ray pulse was assumed as a square wave. It should be noted that the BOI momentum was not directly measured in that experiment. Instead, a stress history $\sigma_{xx}\left(t\right)$ in a sampling point of the panel was recorded during the test. Using Equation (8), the BOI momentum was calculated by a stress integral expressed as:

(29) $$I=\int_{0}^{t_{1}}\sigma_{xx}(t)dt$$

where *t*_1_ is the time of stress transfer. The measured BOI momentum was found to be 440 Pa·s in the experiment. In the present work, the BOI momentum was obtained using Equation (6) by the MBBAY model for comparison. The BOI momentum by Equation (6) was found to be 700 Pa·s with a deviation of 59% of the experimental result in reference [22]. The theoretical solution resulted in a large error in predicting the BOI momentum of the CFRP composite under X-ray. Here, the BOI momentum of the CFRP composite was predicted by our FE model. Using the geometry and material properties described in Section 3, the stress history in a sampling point (*x* = 1/4*H*) in the central axis was calculated as shown in Figure 5. 

**Figure 6:**
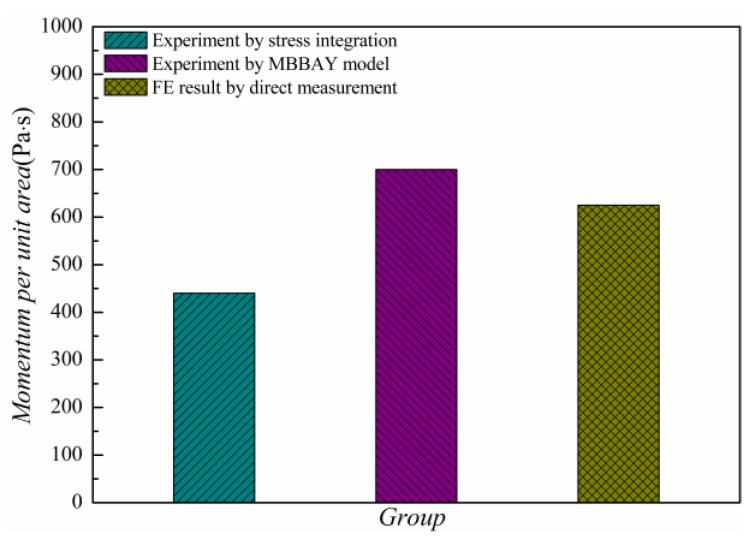
Blow-off impulse (BOI) momentum by experiment and our FE model.

It is seen that the predicted compressive stress increases to a peak of 44.6 GPa within 0.0068 μs and then decreases to zero after 0.0346 μs. The BOI momentum is directly calculated and the value is 625 Pa·s, with the deviation of 42.0% of the experimental result as shown in Figure 6.

Hence, the results predicted by the FE model were closer to the analytical solution. The numerical prediction by our FE model was at the same level as the experimental results, indicating the accuracy of our program. The FE model could thus be used further to determine the dynamic behavior of CFRP composites under X-ray radiation. 

To compare the dynamic behavior of an aluminum panel and the CFRP panel, the stress history and BOI momentum of the CFRP composite were also determined using the material properties described in Section 3.3. Figure 5 shows the stress history of the CFRP target during X-ray radiation. It can be observed that the speed of the stress wave in the CFRP composite is lower than that in the aluminum, whereas the pulse duration is similar. The peak value of the stress in the CFRP material is approximately half of that in the aluminum. In addition, the BOI momentum calculated by Equation (29) is 425 Pa·s, which is 64.7% lower than that in the aluminum. The results showed that the CFRP material effectively reduced the BOI momentum and stress peak value compared to the aluminum under the same X-ray, although the vaporization of the surface materials was unavoidable, as mentioned in Section 2.1.

### 4.2. Dynamic Response ofCarbon Fiber-Reinforced Polymer(CFRP) Composite under Various X-ray Radiations

**Figure 7:**
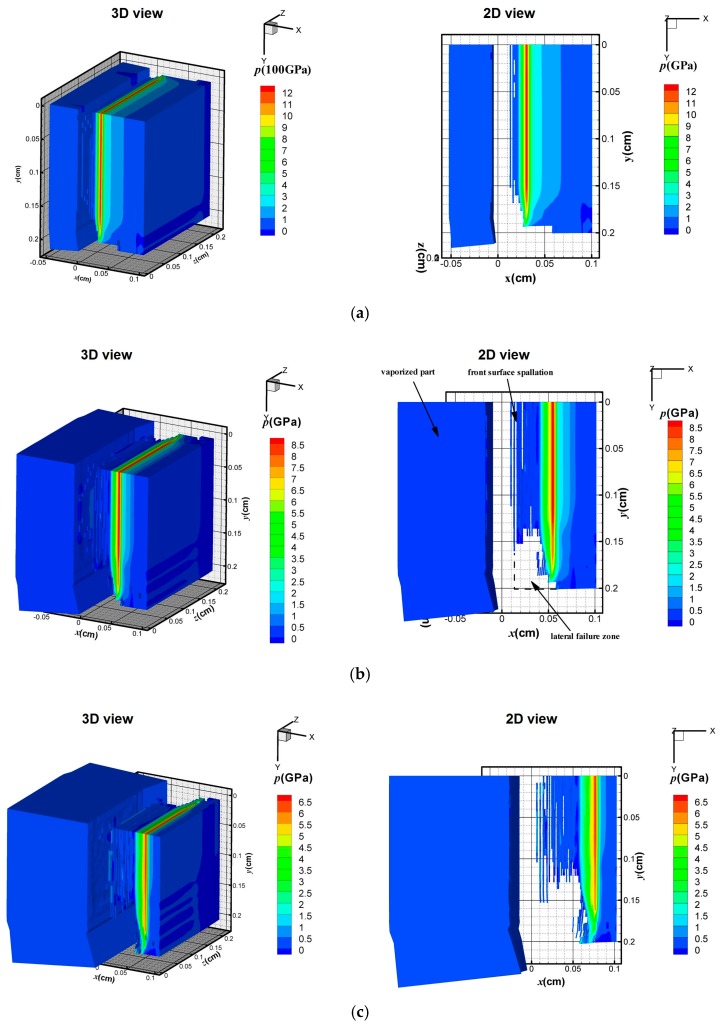
Stress wave under 1 keV X-ray at: (**a**) 0.05 μs; (**b**) 0.10 μs; and (**c**) 0.15 μs.

**Figure 8:**
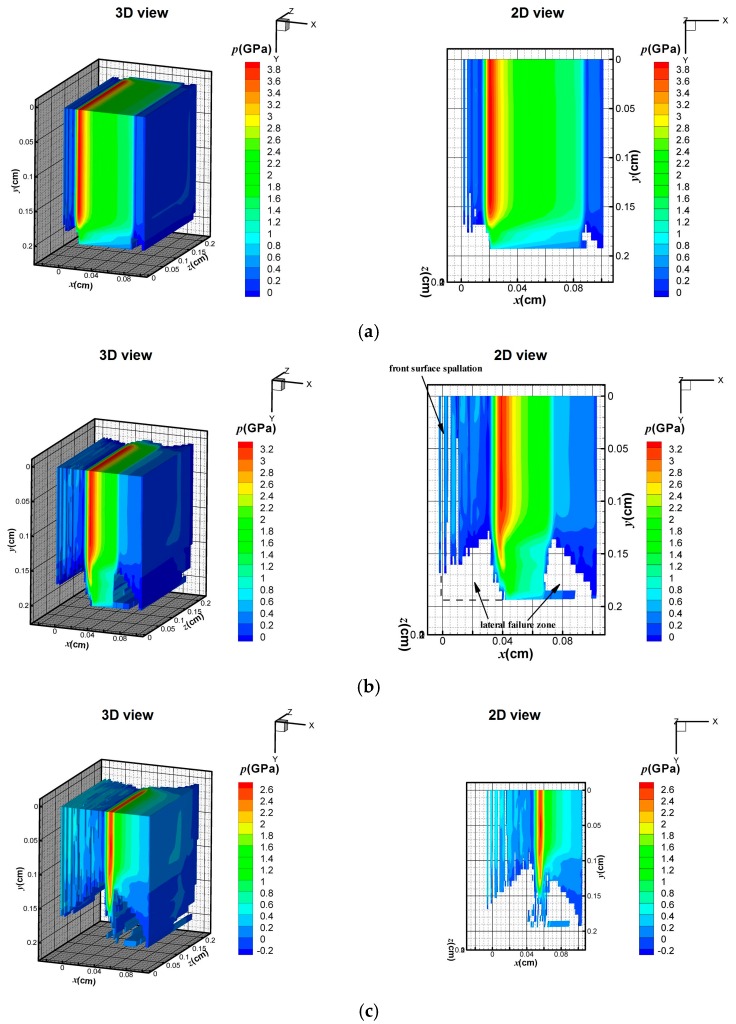
Stress wave under 3 keV X-ray at: (**a**) 0.05 μs; (**b**) 0.10 μs; and (**c**) 0.15 μs.

In this section, the FE model was used to investigate the effect of X-ray with different blackbody temperature (i.e., a soft X-ray *kT* = 1 keV and a hard X-ray *kT* = 3 keV) on the dynamic behavior of the CFRP composite. The waveform and duration of the X-ray pulses were described in Section 4.1. The energy fluence $\Phi_{0}$ was the same in these two X-rays, with a value of 418 J/cm^2^. Figure 7 and Figure 8 show 3D pressure contours and 2D pressure contours of the CFRP composite at *z* = 0 cm at the times of 0.05, 0.10, and 0.15 μs.

**Figure 9:**
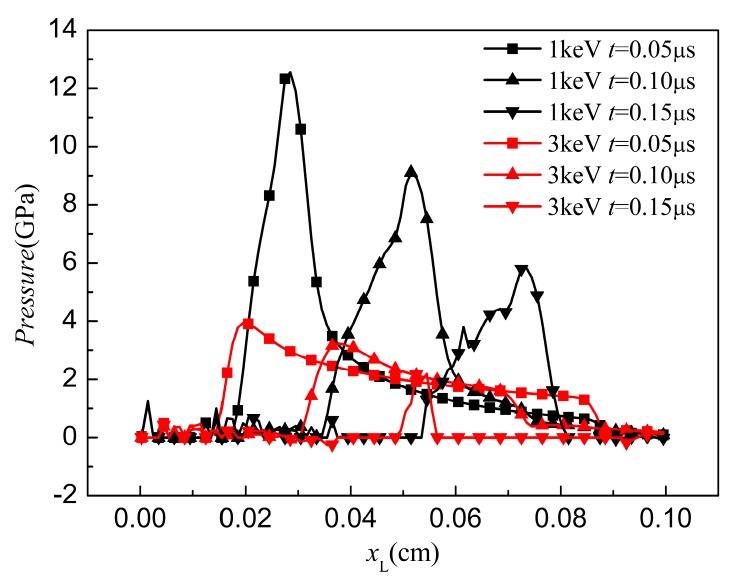
Stress history of CFRP panels.

Although the input energy is the same, the soft X-ray produces surface vaporization in the CFRP composite, as illustrated in Figure 7, whereas no surface vaporization is detected under the hard X-ray, as shown in Figure 8. In Figure 7a, a compressive hydrostatic pressure is generated at *t* = 0.05 μs. The sublimation layer inflates drastically and separates from the residual solid part. The compressive stress wave propagates towards the back surface and the peak value of the stress declines rapidly from 12 to 6.5 GPa within 0.1 μs, as shown in Figure 7a–c. In addition, the elements that meet the maximum tensile stress criterion are deleted from the contours in Figure 7a–c. Front-surface spallation is found in Figure 7b due to the propagation of the tensile stress wave. A similar phenomenon was also predicted by [22] in a one-dimension condition. In our three-dimensional FE model, a lateral rarefaction wave is seen, and elements near the boundary fail, as shown in Figure 7b. Under a 3 keV hard X-ray, Figure 8 shows that no surface vaporization occurs. The magnitude of the compressive stress from 3.8 to 2.6 GPa is lower than that under the 1 keV X-ray. With the propagation of the stress wave, front-surface spallation also occurs, as shown in Figure 8b. Moreover, before the peak stress wave reaches the back surface, a lateral fracture near the back surface area is observed in Figure 8b. At 0.15 μs, the areas of the front-surface spallation and the lateral-fracture zone increase due to propagation of the stress waves. Figure 9 displays the pressure distribution in the CFRP composite along the initial Lagrange coordinates *x*_L_ at different time frames. 

It can be seen that the pressure profiles take a triangular form in all cases. After measuring the pressure peak position at 0.05 µs, the average stress wave speed in 1 keV (5600 m/s) is larger than that in 3 keV (3900 m/s), whereas the magnitude of pressure under 1 keV is higher than that under 3 keV. The peak values of pressure under 1 and 3 keV are 12.5 and 3.9 GPa, respectively. When the time increases to 0.10 µs, the stress wave propagates and the pressure under 1 keV attenuates rapidly. The wave speed is still greater than that under 3 keV. Interestingly, the duration of the pressure under 3 keV significantly declined at *t* = 0.15 µs. 

**Figure 10:**
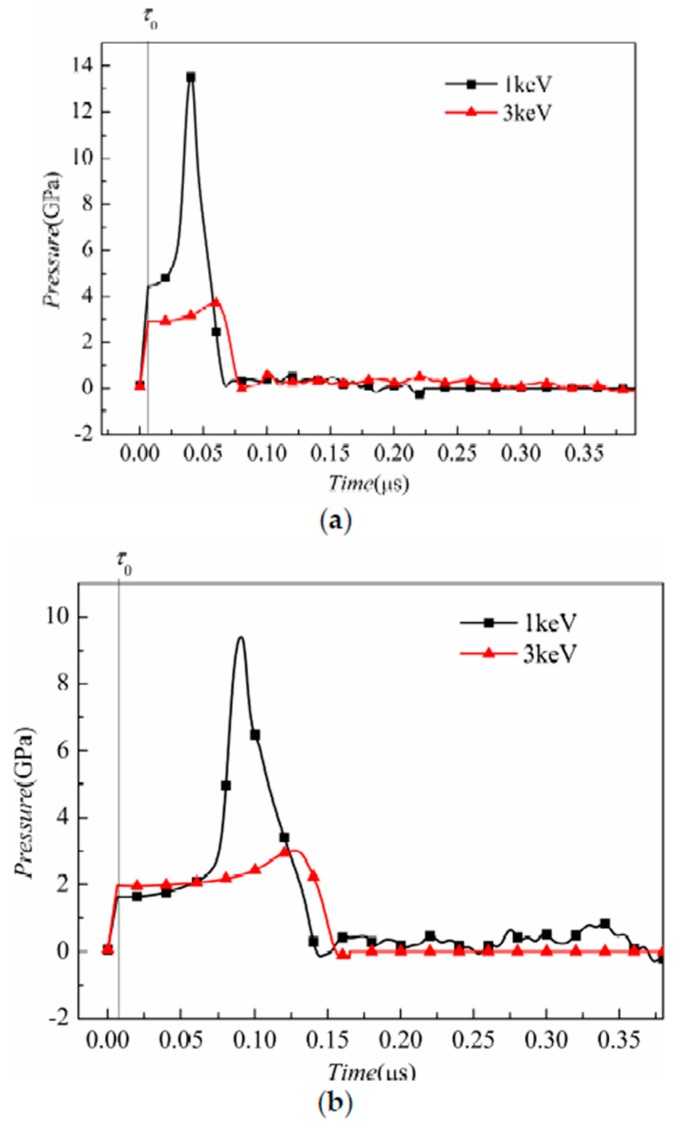

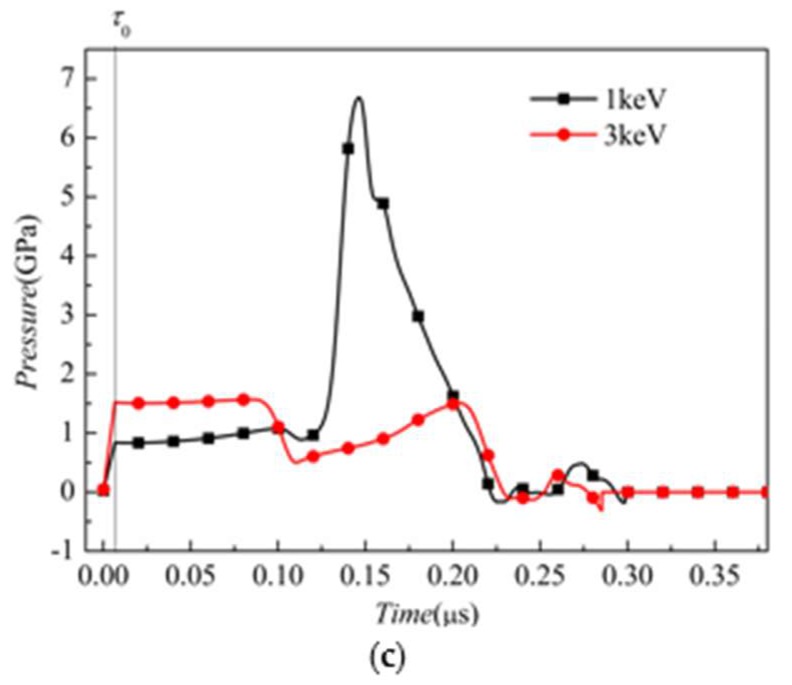
Pressure history at gage points at the distance from the target front surface of (**a**) 1/4*H*; (**b**) 1/2*H*; and (**c**) 3/4*H*.

Furthermore, three sampling points at the center axis of the target are selected to study their pressure history, as shown in Figure 10. 

The distances from the sampling points to the front surface are 1/4*H*, 1/2*H*, and 3/4*H*, respectively. After the radiation duration of $\tau_{0}$, the pressure of each point increases with time and the peak pressure value decreases with the distance from the front surface, as shown in Figure 10a–c. This is because the pressure is induced by the energy deposition. The sampling points nearer to the front surface have higher energy. Because the incident pulse is a square wave, the energy deposition and the corresponding pressure increase linearly with time. In the 1 keV case, the stress-wave propagation leads to an obvious rise in pressure, whereas the pressure is lower in 3 keV case.

**Figure 11:**
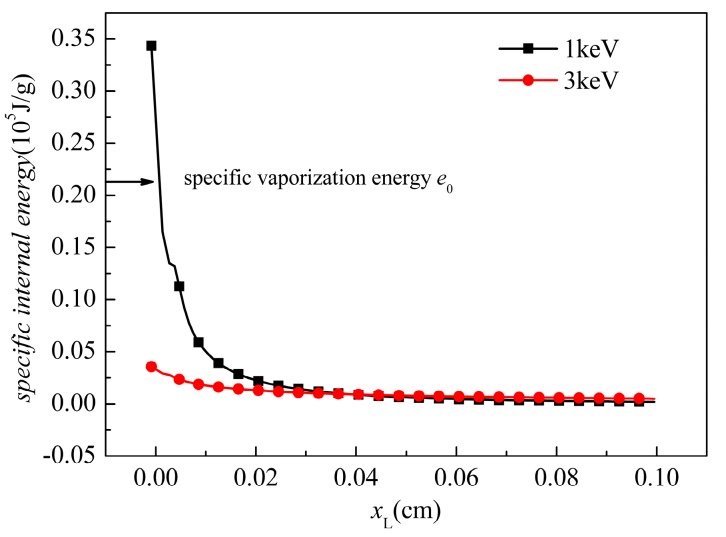
Initial energy distribution after the radiation.

Although the input energy fluences $\Phi_{0}$ are the same in these two simulations, the pressure and the dynamic performance differ. This phenomenon can be explained from the perspective of the specific internal energy distribution. At $\tau_{0}$, after the cessation of radiation, the energy distribution at the central axis is shown in Figure 11. 

The equivalent mass absorption coefficient $\mu_{eff}$ of soft X-ray and hard X-ray were already obtained in Section 2.1. With the soft X-ray (1 keV), the deposition energy attenuates rapidly with the increment of the depth and most of the energy deposits on the surface. Because the deposition energy at the surface elements exceeds the threshold of vaporization, material vaporization occurs. The BOI momentum makes a contribution to the overall stress wave. In the residual solid part in which $x_{L}<0.05\;\mathrm{cm}$, the specific energy maintains a high value, which may generate a correspondingly high thermal stress. Moreover, it should be noted that the tensile strength of the CFRP is in the hundreds MPa level. Therefore, the thermal stress and the lateral rarefaction stress may exceed the tensile strength. In other words, front-surface spallation and lateral fracture can be observed. With the 3 keV X-ray, it is much less than that for the 1 keV case. Because the outermost elements under a 3 keV X-ray do not receive sufficient energy to vaporize, only a thermal stress wave exists. In the material, the specific energy is less than that under a 1 keV X-ray, and thus the peak value of the pressure is lower than that under the 1 keV X-ray. However, in materials where $x_{L}>0.05\;\mathrm{cm}$, deposited energy exists and, therefore, a thermal stress wave propagates in the materials. The compressive stress reflects at the back surface and the lateral free boundary, and the rarefaction destroys the elements. Therefore, both the spallation at the front surface and the fracture near the back surface area occur during the 3 keV X-ray radiation.

## 5. Conclusions

In this work, an in-house Lagrangian explicit three-dimensional FE model with modified PUFF EOS was developed to study the dynamic and damage behavior of CFRP composites under X-ray radiation. The following conclusions can be drawn:

(1) The FE prediction of BOI momentum in an aluminum panel was at the same level as the experimental result, indicating the effectiveness of the FE model.
(2) Compared to the aluminum panel, the CFRP panel effectively reduced the BOI and peak stress under the same radiation condition.
(3) The FE results revealed that 1 keV X-ray resulted in surface vaporization, whereas no surface vaporization was seen when 3 keV X-ray was used, although front-surface spallation was seen in both cases. Furthermore, the magnitude of the stress wave under 1 keV X-ray was higher than that under 3 keV X-ray.

Currently, the main challenge is the computational efficiency of the FE program. The FE program we developed was compiled by the Compaq Visual Fortran 6.6 compiler. For one case analysis in this work, the run-time cost was 12–16 h in a single desktop computer (CPU number: 16). In future, we are planning to improve the computational efficiency by using a parallel MPI protocol to simulate a full-size radiation problem.

## 6. Patents

In this work, the results are simulated by our in-house program: “X she xian san wei re-li xue xiao ying mo ni ruan jian” (Kun Zhang, Wenhui Tang, Xianwen Ran. CN. patent number: 2016SR110024).

## Figures and Tables

**Table 1:** Material properties of CFRP composite.

Elastic Parameters		Plastic Parameters		EOS Parameters		Failure Parameters	
*E*_11_ (GPa)	9.7	*a*_11_	0.669	*K*′ (GPa)	25.04	*σ*_11_ (GPa)	0.2457
*E*_22_ (GPa)	72.9	*a*_22_	0.025	$\Gamma_{0}$	1.098	*σ*_22_ (GPa)	0.6190
*E*_33_ (GPa)	22.89	*a*_33_	1	*s*	1.049	*σ*_33_ (GPa)	0.1950
*v*_12_	0.0187	*a*_12_	0	*γ*	1.667	*σ*_12_ (GPa)	0.0475
*v*_13_	0.218	*a*_13_	−0.471	Estimated *c*_0_ (m/s)	4003	*σ*_13_ (GPa)	0.0285
*v*_23_	0.77	*a*_23_	−0.128	*ρ*_0_ (g/cm^3^)	1.563	*σ*_23_ (GPa)	0.0393
*G*_12_ (GPa)	0.873	*a*_44_	0.061	Estimated *e*_0_ (kJ/g)	21.5		
*G*_13_ (GPa)	0.558	*a*_55_	3.157				
*G*_23_ (GPa)	48.35	*a*_66_	2.128				

**Table 2:** Material properties of aluminum.

Elastic Parameters		Plastic Parameters		EOS Parameters		Failure Parameters	
*E* (GPa)	71.71	*k* (GPa)	0.5	*K* (GPa)	78.73	*σ* (GPa)	1.2
*v*	0.33			$\Gamma_{0}$	2.18		
*G* (GPa)	27.1			*s*	1.35		
				*γ*	1.667		
				*c*_0_ (m/s)	5400		
				*ρ*_0_ (g/cm^3^)	2.7		
				*e*_0_ (kJ/g)	13.5		
